# Comparison of divergent breeding management strategies in two species of semi-captive eland in Senegal

**DOI:** 10.1038/s41598-020-65598-6

**Published:** 2020-06-01

**Authors:** Anna Kubátová, Kateřina Štochlová, Karolína Brandlová, Pavla Jůnková Vymyslická, Barbora Černá Bolfíková

**Affiliations:** 1 Department of Animal Science and Food Processing, Faculty of Tropical AgriSciences, Czech University of Life Sciences Prague, Prague, Czech Republic; 2 Department of Ecology, Faculty of Environmental Sciences, Czech University of Life Sciences Prague, Prague, Czech Republic

**Keywords:** Conservation biology, Molecular ecology, Animal breeding

## Abstract

Breeding management of small populations may have a critical influence on the development of population characteristics in terms of levels of genetic diversity and inbreeding. Two populations of antelope sister species – Critically Endangered Western Derby eland (*Tauroragus derbianus derbianus*) and non-native Least Concern Cape eland (*Taurotragus oryx oryx*) bred under different management strategies were studied in Senegal, Western Africa. The aims of the study were to compare the population genetic parameters of the two species and to test for the presence of interspecific hybrids. In total, blood and tissue samples from 76 Western Derby elands and 26 Cape elands were investigated, using 12 microsatellite markers. No hybrid individuals were detected in the sampled animals within the multispecies enclosure in Bandia Reserve, Senegal. The parameters of genetic polymorphism indicated much lower genetic diversity in Western Derby elands compared to Cape elands. On the other hand, the coefficient of inbreeding was low in both species. It is hypothesized that this could be a positive effect of strict population management of Western Derby elands, which, despite the loss of genetic diversity, minimizes inbreeding.

## Introduction

Genetic diversity represents an essential pillar for the survival of populations through the possibility of adapting to a changing environment. Problems connected with maintaining diversity are common in captive populations, whose sizes are limited by space and the individuals are scattered among institutions worldwide so the gene flow is restricted^1–3^.

To eliminate the negative impact of the phenomena affecting small populations (e.g. loss of variability due to genetic drift and inbreeding), it is necessary to apply appropriate genetic management^4,5^, which can vary from intensive interventions^6,7^ to simple monitoring^8^. However, for the decision-making process, it is essential to know some basic information about the kinship and genetic variability of the individuals^9^ which is usually recorded in studbooks. Even if the studbooks for captive wildlife exist, for example, European studbooks^10^ or International studbooks^11^, knowledge of kinship across these populations is often limited and can be as low as only 50% or less in some ungulate species kept in zoos^12^.

However, there are also species with better background information recorded, where a high proportion of their pedigree is known (% PK). These are mainly endangered species for which special *ex situ* conservation programmes have been created, such as the European Endangered Species Programmes (EEPs) for species like the Cuvier’s gazelle (*Gazella cuvieri*, 100% PK)^13^, or the recently established EAZA *Ex situ* Programmes^10^.

Pedigree data are especially valuable in the evaluation of ancestry and kinship^14^ but even the studbooks do not guarantee reliable information concerning genetic parameters of polymorphism within the populations. The reason for the lack of reliable genetic data is mostly because the studbook analyses rely on the assumption that the founders are unrelated and non-inbred, which is not always the case^6,15^. Only genetic monitoring can reveal the true genetic polymorphism of populations^16^, including the ones without studbooks^8^.

Loss of genetic variability is not the only issue of genetics in captive breeding of wildlife. The populations can face problems with interspecific hybridization, which is considered as a very important factor that endangers biodiversity and the existence of many species^17^. Even if the hybridization occurs in nature, it can also be caused by human interference, usually by keeping related, but initially geographically isolated, taxa together; for example, in wildlife reserves or mixed-species exhibits in zoos, which is currently very popular^18–20^.

Small isolated populations of two eland species with different conservation statuses and different geographical origins are kept in Bandia Reserve in Senegal. Both populations had a similar number of founding animals, but they have been managed differently and thus may serve as an ideal model for evaluation of the influence of population management on the genetic parameters of the population.

Western Derby elands (WDE, *Taurotragus derbianus derbianus*) are represented worldwide by less than 200 free-ranging individuals in Senegalese Niokolo Koba National Park (NKNP)^21^. A unique conservation programme for this antelope takes place in two Senegalese wildlife reserves, Bandia and Fathala, where about 100 of these antelopes live in semi-captive conditions^22^. When their founders were captured in NKNP in 2000, the total WDE population was already very limited and WDE was considered as Endangered. The population in NKNP was estimated to total only around 100 individuals at that time^23^ and all the founders of the captive programme were captured from just one herd^24^. The founders were transported to Bandia Reserve where the first captive breeding of WDE started. In 2006, the primary selected animals from Bandia Reserve were transported to Fathala Wildlife Reserve^25^.

According to the WDE Conservation Strategy, the conservation programme’s “…aim is to manage the population to retain as high as possible genetic diversity…”^26^. For this purpose, the WDE semi-captive population is intensively managed to minimize kinship since its establishment^24^ via annual identification of new-born calves and their mothers through suckling observations for studbook creation^27^, transportation of sub-adult offspring to other breeding herds to avoid backcrossing^28^, and quite recently even genetic monitoring to compare pedigree and microsatellite data^6^. However, natural gene flow between the reserves and the NKNP does not currently exist and the inbreeding rate of the population is increasing while the polymorphism is dropping rapidly^6^. On the other hand, Cape elands (CE, *Tauroragus oryx oryx*) were introduced to Bandia Reserve in 1996 to increase its attractiveness for safaris for visitors. The only management applied in the population of CE in Bandia Reserve involves the culling of surplus males and older calf-less females for meat production. Since the initiation of both breeding programmes, no animals have been imported into the populations of WDE and CE. For a comparison of the background of both studied populations, see the overview in Table 1.

**Table 1:** Overview of the background of the Western Derby eland (WDE) and Cape eland (CE) populations living in Bandia Reserve, Senegal.

Characteristic/Population	WDE	CE
Origin of animals	Native Senegalese fauna	Introduced species
Conservation status	Critically Endangered	Least Concern
Population establishment	2000	1996
Source of the founders	Niokolo Koba National Park	South Africa
Number of founders	1 male: 5 females	3 males: 5 females
Population monitoring	Monitored (studbook publications, genetic monitoring)	No monitoring so far
Genetic management	Managed (1 bachelor and 5 reproductive herds, animal transports between herds, no culling)	Unmanaged (one herd, random males and recently even old diseased females culled for meat purposes)
Number of individuals	101 (June 2017, including the stock in Fathala Wildlife Reserve)	200–250 (February 2017, rough estimate of the Director for animals)
Resources	Brandlová *et al*.^22,26^, Zemanová *et al*.^6^, IUCN^45^	IUCN^46^, Bandia Reserve veterinary records unpubl. data

In Bandia Reserve, all WDE were kept in fenced areas, separated from other species, until July 2012 when reserve managers decided to remove some of the fences in the reserve, and thus two previously separated breeding herds of WDE were merged and mixed with other animal species. Since that time, this WDE herd has been in physical contact with CE^26^. The length of pregnancy in the WDE is approximately 9 months^25^, so all WDE offspring which have been born in the multispecies enclosure in Bandia Reserve since April 2013 should be considered as potential hybrids, in other words, 26 potential hybrids were born up from this period until June 2017^22^.

Even though a hybrid of the Derby eland has never been observed, there is a risk of its hybridization with CE in Bandia Reserve, considering the previous experience with other Tragelaphini antelopes that are characterized by “unusual readiness to hybridize in captivity”^19,29–32^. Such hybridization would jeopardize the entire conservation programme and thus there is an urgent need for monitoring, as well as genetic variability assessment, and subsequent evaluation of the appropriateness of the applied population management.

The aims of this study were to:

1. Test for the presence of interspecific hybrids between semi-captive Western Derby elands (*T. derbianus derbianus*) and Cape elands (*T. oryx oryx*) living in a multispecies enclosure in Bandia Reserve, Senegal;
2. Compare the population genetic parameters of the two divergently managed species and evaluate the effect of the applied population management.

## Materials and Methods

### Sampling

Individuals of two semi-captive populations were included in the study: WDE (n = 76) from Bandia and Fathala reserves in Senegal, and CE (n = 26) from Bandia Reserve, Senegal.

Blood and tissue samples of WDE were acquired systematically by continuous whole population sampling, and included all living individuals recorded in the studbook from 2017^22^ with the exception of 25 animals, from which the samples were not available for various reasons (too young age, absence of veterinarian at the time of observation, etc.). Samples from CE were obtained by random sampling of live calves at the age of 1–3 years, animals that died naturally, and surplus males and older calf-less females culled for meat production by the staff of Bandia Reserve.

All samples were collected in Senegal in the period 2005–2017 by the NGO Derbianus Conservation (DC, formerly Derbianus Czech Society for African Wildlife) in cooperation with the Directorate of National Parks of Senegal (DPN) and Society for the Protection of Environment and Fauna in Senegal (SPEFS), who are recognized on an international level (Memorandum of Understanding between the Ministry of the Environment of the Czech Republic and the Ministry of the Environment of Senegal; Implementation Agreement to the Memorandum between the Ministry of the Environment of the Czech Republic, DC and the Czech University of Life Sciences Prague; Trilateral Agreement between DC, DPN, and SPEFS). The samples were then imported to the Czech Republic (requested veterinary conditions for import with reference numbers SVS/2210/2012-ÚVS, SVS/2015/007838-G and SVS/2017/032757-G have been fulfilled) and provided by the NGO for research purposes. No animal was sacrificed for this study. All sampling procedures were performed by certified veterinarians, except the collection of tissues from dead animals. Blood and hair samples were collected only from anesthetized WDE individuals during transport between breeding herds and the reserves, which regularly occurs because of the genetic management of the population^25^. Tissue samples were collected from both species either from dead individuals by scalpel or from living unanesthetized animals by Biopsy & DNA darts Pneu-Dart, Inc.

Immediately after collection, the hair and tissue samples were fixed in 96% ethanol, and the blood samples were treated with anticoagulants. Then, all the samples were transported to the freezers in the shortest possible time to be stored at −20 °C until processing in a laboratory which has approval (No CZ 11712934) for storage and usage of animal material according to § 48(1)(i) of Act No 166/1999s concerning veterinary care and amending certain related laws, as amended, pursuant to Article 17(1) of Regulation of the European Parliament and the Council (EC) No 169/2009 and Article 27(1) of Commission Regulation (EU) No 142/2011.

### Processing of the samples

Genomic DNA from hair and tissue samples was extracted by Qiagen^®^ DNeasy^®^ Blood & Tissue Kits. In the case of blood samples, Geneaid^™^ Genomic DNA Mini Kits (Blood-Cultured Cell) were used. Twelve microsatellite loci were selected for the analyses because microsatellites are fast evolving markers suitable for analyses of recent population structure^33^.

The PCRs were carried out in T100^™^ Thermal Cyclers from Bio-Rad using the Qiagen^®^ Multiplex PCR kit according to the enclosed protocol^34^. During reactions, 12 microsatellite loci were amplified with already published fluorescently labelled primers that were chosen based on cross-species amplification, including 5 previously used for WDE^6^; for details see Table S1 in Supplementary Material. Fragmentation analyses was performed using a 3500 Genetic Analyzer (Applied Biosystems^™^) with the GeneScan™ 500 LIZ™ dye Size Standard.

### Data analysis

Data from the fragmentation analyses containing information about allele lengths of individual loci were manually scored using GeneMarker^®^ Version 2.2.0, SoftGenetics LLC^®^^35^ and then binned by AutoBin^36^. Large allelic dropout, presence of stutter bands, and null alleles were tested in Micro-Checker 2.2^37^. Departures from the Hardy–Weinberg equilibrium and linkage equilibrium of all microsatellite loci were checked by Genepop 4.2^38^.

Population structure and the presence of hybrids were tested by the Bayesian clustering method in Structure 2.3.4^39^ with 1,000,000 steps of Markov Chain Monte Carlo repetitions after 100,000 steps of the burn-in period. The analysis was run for 1–5 clusters (*K*) always with 5 repetitions for each *K*. The results were post-processed by Structure Harvester to evaluate the best supported K^40^.

Factorial correspondence analysis was done in Genetix 4.05^41^. To compare the populations of WDE and CE, basic population structure characteristics were obtained using different programs intended for population genetics calculations. Values of observed (Ho) and expected (He) heterozygosity were calculated in GenAlEx 6.502^42^, inbreeding coefficient (F_IS_), fixation index (F_ST_) and allelic richness (Ar) were obtained via FSTAT 2.9.3.2^43^, which was also used for the counting of the confidence interval of F_ST_. The confidence interval of F_IS_ was evaluated using Genetix 4.05.

## Results

In total, 76 samples of WDE and 26 samples of CE were genotyped. Analysis in Micro-Checker 2.2 did not detect any genotyping errors, including the presence of null alleles. Linkage disequilibrium was not detected. The populations were in Hardy–Weinberg equilibrium at all of the studied loci, except CE at ETH10 (p = 0.025) and WDE at CSRM42 (monomorphic locus in WDE).

**Figure 1:**
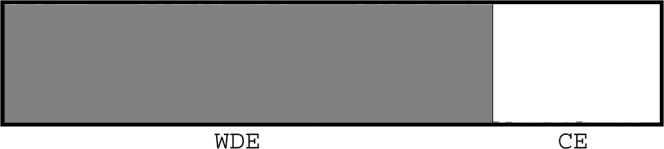
Results from Structure showing assignment of the tested individuals (n = 102) into two clusters corresponding with their species – Western Derby eland (WDE, n = 76) and Cape eland (CE, n = 26).

The highest likelihood was obtained for two clusters (see Fig. S1 in Supplementary material). Each animal was correctly assigned to its presumptive species. The analysis did not reveal any signal of recent hybridization between the species (Fig. 1).

**Figure 2:**
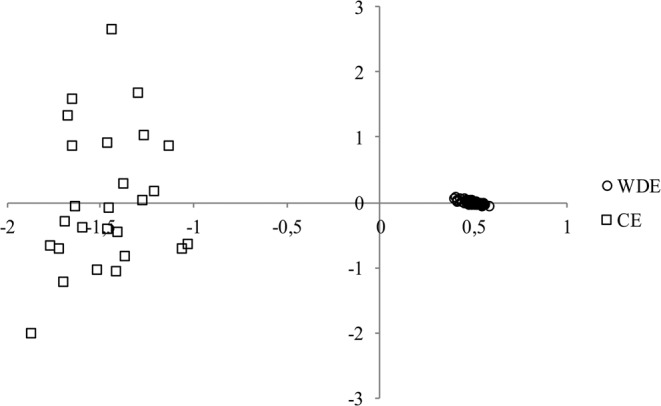
Results of the factorial correspondence analysis from Genetix based on 12 microsatellite loci showing a multivariate relationship between individuals of Western Derby elands (WDE, n = 76) and Cape elands (CE, n = 26).

Factorial correspondence analysis visualized the relationships between all tested individuals of the tested populations (Fig. 2). Western Derby elands are represented by a homogenous cluster with little variation compared with CE.

The results of the selected population structure characteristics to compare the populations of WDE and CE are presented in Table 2, and show higher heterozygosity and genetic diversity in CE than in WDE. However, both species have a low level of inbreeding.

**Table 2:** Values of basic population characteristics counted for both tested populations – Western Derby elands (WDE) and Cape elands (CE).

Characteristic/Population	WDE (n = 76)	CE (n = 26)
He	0.425	0.755
Ho	0.445	0.771
Ar	2.462	5.873
Polymorphism	0.917	1.000
F_IS_ (95% confidence interval)	−0.046 (−0.061–0.003)	−0.021 (−0.096–0.003)
F_ST_ (95% confidence interval)	0.361 (0.295–0.429)	

## Discussion

Derby elands (*Taurotragus derbianus*) and common elands (*Taurotragus oryx*) are considered as sympatric only in South Sudan^44–46^. So far, there is no information about the occurrence of hybridization between them, but it cannot be excluded, due to the high relatedness of the taxa (separation estimated at 1.6 million years before present)^47^ and due to the presence of hybrids between other Tragelaphini species^19,29–32^. The present study did not detect any individual of hybrid origin. However, not all suspect individuals were sampled and thus some possible hybrids might have been overlooked. In the WDE population, 16 samples from totally 26 suspect animals were tested while three of them have died before being sampled and never reproduced. Moreover, samples were taken randomly in the CE population. It may be concluded that until June 2017, no hybrid was detected, and considering this, the probability of ongoing hybridization is low. However, the risk of hybridization between eland species in Bandia Reserve still exists, as the species remain in direct contact^48^. Also, one cannot exclude that post-zygotic reproduction isolating mechanisms may exist, and that the embryo may be lost during its development. Should this be the case, the fitness of the whole herd would decrease. Respecting the uniqueness and conservation status of the WDE, we propose continuous monitoring of hybridization between WDE and CE until they have separated breeding facilities. If the hybrids occur, it may have severe consequences for the genetic integrity of the species, as shown i.e. in the giant sable antelope (*Hippotragus niger variani*)^49^ and bontebok (*Damaliscus pygargus pygargus*)^50^.

Relatedness of the individuals and the sex ratio within the founding herd highly affected the genetic diversity of the studied populations. Parameters of genetic polymorphism (Ar, Ho) are much higher in CE (Table 2). Factorial correspondence analysis also supports higher variation within CE (Fig. 2). Two important factors should be considered: firstly, there is an assumption that the founders of the WDE semi-captive population were already related^6^. The second important factor to consider is the presence of just one male founder of the WDE semi-captive population^25^, and thus the level of inbreeding within WDE increased rapidly over the generations^6^. In CE, the sex ratio of the founders was more balanced, containing three males and five females^51^. Although dominant eland males are usually considered as sires of all the offspring in their herds^25^, this is not necessarily true considering the studies in other ungulate species^52,53^.

Genetic management of the Critically Endangered WDE might positively affect the level of inbreeding of the whole population in semi-captivity. The values of the coefficient of inbreeding (Table 2) are comparable over the species, even though the other parameters of polymorphism are much lower in WDE (Table 2). Nevertheless, even though microsatellites were shown to be useful tools for the study of the genetic diversity of ungulates^54^, they have their limitations^55^, which must be considered regarding the results. They do not reflect the whole genome and therefore provides only partial information about the level of polymorphism, and often it is impossible to correlate specific traits with microsatellite parameters.

The genetic background of the population should always be considered from the onset of the establishment of a conservation programme. In optimal circumstances, there are more suitable candidate populations in the wild from which intended founders of the backup population can be selected. All founders should ideally be unrelated and their numbers sufficient to establish more breeding pairs to avoid kinship in the first generation^6,16,56^. However, this is not always possible in the case of Critically Endangered species, in which animal numbers have decreased to such an extent that backup breeding programmes must be established with only a few remaining founders^57^. An example of an extreme situation is the attempt to save the subspecies of northern white rhinoceros (*Ceratotherium simum cottoni*) via hybridization with the conspecific southern white rhinoceros (*Ceratotherium simum simum*), despite their natural long period of geographic, and thus genetic, isolation^58^. Nevertheless, there should be always an effort to avoid the possibility of both inbreeding and outbreeding depression, if possible^16^.

Mitigating genetic threats of small isolated populations, such as inbreeding, and the negative consequences associated with the loss of genetic diversity can involve conservation actions such as translocations^8^. Even though such actions can be risky either from the epidemiological or genetic point of view^59^, they may be crucial for species survival if managed properly^60^. In the case of the WDE semi-captive population, further importation of new individuals from NKNP to reduce inbreeding and increase genetic diversity has been already recommended^6,25^. The results of the present study regarding the genetic diversity of the WDE semi-captive population are in accordance with these previous conclusions and support the idea of introducing new founders from the wild. It also corresponds with the recommendations of Ochoa *et al*.^61^ to promote mutual and continuous gene flow via translocations between populations in the wild and captive populations which should function as a source of genetic variation for reintroduction programmes. The One Plan Approach as an integrated approach to species conservation consisting of management strategies and conservation actions by all responsible parties for all populations of a species, whether inside or outside their natural range, should be a priority^62,63^.

However, in the case of WDE, the promotion of gene flow is needed not only between the wild and captive populations, but also within the captive population. Otherwise, they could suffer from high differentiation as was described in the subpopulations of Arabian oryx^61^. Although the WDE semi-captive population was considered as a whole in the present study, the individuals are kept in two separate reserves, and regular animal transport takes place between the breeding herds, mostly within each reserve. The transport of animals from Bandia Reserve to Fathala Wildlife Reserve took place in 2006, 2008, 2009 and 2011, with a total of 32 individuals (22 males and 10 females) being translocated and organized into one bachelor herd at first, and later, two breeding herds^25^. Since only a limited number of females succeeded in reproducing in Fathala Wildlife Reserve, the population in Fathala suffered multiple founder effects, and pedigree analysis suggests considerably lower genetic diversity in Fathala Wildlife Reserve than in Bandia Reserve^1–3,64^.

Wespi *et al*.^7^ concluded that population management including interventions possibly influencing genetic diversity via regular changes of breeding males does not always offer distinct advantages when compared to unmanaged populations. However, their study did not reflect genetic parameters. In contrast, the results of the present study indicates that genetic management could have a positive effect on the genetic background of populations. Considering the results of this and a previous study by the present authors^6^, it may be concluded, that for highly inbred populations such as WDE, genetic management keeps inbreeding at a low level, despite low genetic polymorphism and high relatedness of the population.

## Supplementary information

Supplementary info.

## Data Availability

Upon acceptance of the manuscript, genotypes used in the final analyses will be deposited at Dryad Digital Repository.
