# Supplementary material for: Comparison of divergent breeding management strategies in two species of semi-captive eland in Senegal
Source: Sci Rep. 2020 Jun 1;10:8841. doi: 10.1038/s41598-020-65598-6 (PMC7264215; doi:10.1038/s41598-020-65598-6)
Supplement: Supplementary file 1 — Supplementary info. [file 41598_2020_65598_MOESM1_ESM.docx]

Comparison of divergent breeding management strategies in two species of semi-captive eland in Senegal.

Anna Kubátová^1^, Kateřina Štochlová^1^, Karolína Brandlová^1^, Pavla Jůnková Vymyslická^2^, Barbora Černá Bolfíková^1,*^

^1^ Department of Animal Science and Food Processing, Faculty of Tropical AgriSciences, Czech University of Life Sciences Prague, Prague, Czech Republic

^2^ Department of Ecology, Faculty of Environmental Sciences, Czech University of Life Sciences Prague, Prague, Czech Republic

* Corresponding author

E-mail: [bolfikova@ftz.czu.cz](mailto:bolfikova@ftz.czu.cz)

ORCID:

0000-0001-8059-4889 (BČB)

0000-0002-2014-7275 (KB)

**Supplementary material**

Table S1: List of 12 microsatellite primers in the form of three primer mixes that were used in the study with the annealing temperature 58 °C:

| primer mix 1 | BL42 [^64^], BRR [^65^], CSRM60 [^65^], ETH10 [^65^], ETH225 [^6^], X800214 [^6^] |
| --- | --- |
| primer mix 2 | BM4505 [^6^], CSSM42 [^66^], INRA107 [^66^], SPS113 [^66^] |
| primer mix 3 | AF533518 [^6^], OarFCB304 [^6^] |

[6] Zemanová, H., Černá Bolfíková, B., Hulva, P., Hejcmanová, P. & Brandlová, K. Conservation genetics of the Western Derby eland (T*aurotragus derbianus derbianus)* in Senegal: Integration of pedigree and microsatellite data. *Mammalian Biology* **80**, 328-332 (2015).

[65] Bishop, M. D. *et al.* A Genetic-Linkage Map for Cattle. *Genetics* **136**, 619-639 (1994).

[66] Fernandez, M. E. *et al.* Comparison of the effectiveness of microsatellites and SNP panels for genetic identification, traceability and assessment of parentage in an inbred Angus herd. *Genetics and Molecular Biology* **36**, 185-U194, doi:10.1590/s1415-47572013000200008 (2013).

[67] Slate, J. *et al.* Bovine microsatellite loci are highly conserved in red deer (*Cervus elaphus*), sika deer (*Cervus nippon*) and Soay sheep (*Ovis aries*). *Animal Genetics* **29**, 307-315, doi:10.1046/j.1365-2052.1998.00347.x (1998).

A B


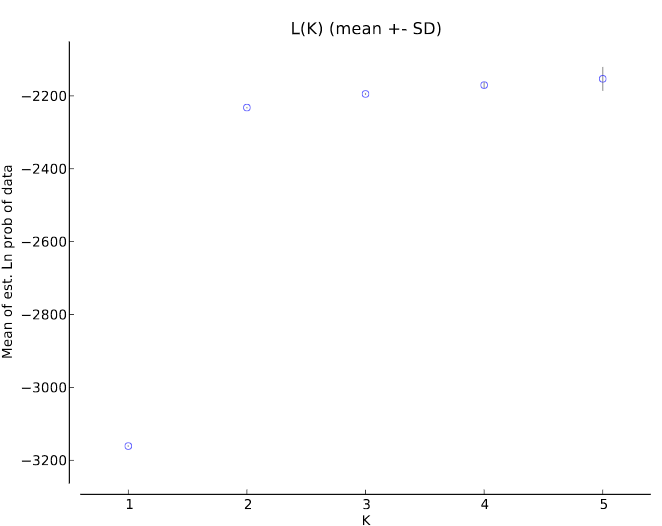

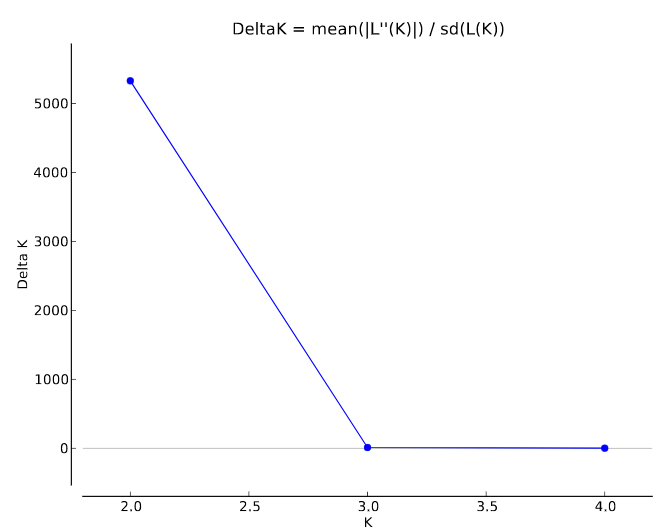


A2

Fig. S1: Plots generated from Structure Harvester. A: Mean likelihood L(K) and variance per K value from Structure on a dataset containing 102 individuals genotyped for 12 microsatellite loci. B: Evanno’s delta K statistic plot detecting the number of K groups that best fit the data, here 2 clusters.
